# The Learning Curve of Da Vinci Robot-Assisted Hemicolectomy for Colon Cancer: A Retrospective Study of 76 Cases at a Single Center

**DOI:** 10.3389/fsurg.2022.897103

**Published:** 2022-06-29

**Authors:** Pu Huang, Sen Li, Peng Li, Baoqing Jia

**Affiliations:** ^1^ Medical School of Chinese PLA, Beijing, China; ^2^ Department of General Surgery, the First Medical Centre, Chinese PLA General Hospital, Beijing, China

**Keywords:** robotic surgery, colon cancer, right hemicolectomy, learning curve, cumulative sum (CUSUM)

## Abstract

**Background and Aims:**

Robotic-assisted right hemicolectomy (RARH) has many benefits in treating colon cancer, but it is a new technology that needs to be evaluated. This study aims to assess the learning curve (LC) of RARH procedures with the complete mesoscopic exception and D3 lymph node dissection for colon carcinoma.

**Methods:**

A retrospective analysis was performed on a consecutive series of 76 patients who underwent RARH from July 2014 to March 2018. The operation time was evaluated using the cumulative sum (CUSUM) method to analyze the LC. The patients were categorized into two groups based on the LC: Phase I and Phase II. Statistical methods were used to compare clinicopathological data on intraoperative and perioperative outcomes at different stages of the study.

**Results:**

The peak point of the LC was observed in the 27th case. Using the CUSUM method, we divide the LC into two stages. Stage 1 (initial learning stage): Cases 1–27 and Stage 2 (proficiency phase): Cases 28–76. There were no obvious distinctions between the two patients’ essential characteristics (age, sex, body mass index, clinical stage, and ASA score). The mean operation time of each group is 187.37 ± 45.56 min and 161.1 ± 37.74 min (*P* = 0.009), respectively. The intraoperative blood loss of each group is 170.4 ± 217.2 ml and 95.7 ± 72.8 ml (*P* = 0.031), respectively.

**Conclusion:**

Based on the LC with CUSUM analysis, the data suggest that the learning phase of RARH was achieved after 27 cases. The operation time and the intraoperative blood loss decrease with more cases performed.

## Introduction

Colorectal cancer (CRC) remains a global health threat as a malignant tumor and the fourth leading cancer by incidence and mortality worldwide (1). Currently, laparoscopic surgery is a standard treatment for CRC. Compared with the open approach, it has a similar oncological outcome and faster recovery. Also, the safety and efficacy of the procedure have been demonstrated in many randomized controlled trials (RCTs) (2). However, laparoscopic technology also has some shortcomings, such as the tremor produced by human muscles, insufficient flexibility, and loss of 3D vision. Some surgeons believe that the robotic approach could address many of the limitations of standard laparoscopic colorectal surgery (3). Consequently, there has been a steady increase in the adoption of the robotic technique in colorectal surgery (4).

As a new surgical technology, the robotic surgical system has changed the way surgeons perform operations. It is becoming available in more and more hospitals and is being used to treat CRC. Several studies have shown that robotic colorectal cancer surgery is safe and feasible compared with laparoscopic surgery (5–7). But the robotic system may have unique advantages, including improved depth perception, improved dexterity and control, and improved ergonomics for surgeons (8). The number of robotic colorectal cancer surgery is on the rise, and this system is being used in the treatment of right colon cancer. The learning curve (LC) reflects how a skill is acquired over time. Previous experience in laparoscopic colorectal surgery can minimize the LC for the same type of robotic surgery (9). Laparoscopic experience may also influence the LC of robotic radical right colon surgery.

Our research team has so far performed more than 3,000 gastrointestinal laparoscopic surgeries, accumulating a lot of experience in minimally invasive surgery, and has been conducting trials on robot-assisted right hemicolectomy for colon cancer since 2014. This study retrospectively analyzed oncology patients undergoing robotic-assisted right hemicolectomy (RARH) operated by the same operator from July 2014 to March 2018. The data indicators related to the perioperative phase were analyzed by the cumulative sum (CUSUM) method, and LCs were plotted to provide a reference for the conduct of future procedures.

## Methods

We report a retrospective-based study of patients who underwent right hemicolectomy using the da Vinci Si system between July 2014 and March 2018. Exclusion criteria: (I) patients undergoing palliative surgical resection; (II) patients with a combination of severe cardiopulmonary, hepatic and renal organic pathology that is difficult to correct in the short term and cannot tolerate their pneumoperitoneum; (III) patients undergoing emergency surgery; and (IV) patients with multiple primary CRCs. Therefore, all patients were diagnosed by colonoscopic pathological biopsy and signed consent for surgery according to the medical standard. According to the above criteria, 76 patients were finally classified in this study. Patient data included ASA score (American Society of Anesthesiologists), age, body mass index (BMI), operative time, intraoperative bleeding, pre-operative clinical staging, length of hospital stay, Charlson Comorbidity Index (CCI), and pathological findings. The primary aim was to assess the surgical robot's feasibility, safety, and clinical outcomes.

The surgeon included in the study performed all of the robotic-assisted right hemicolectomies using extracorporeal anastomoses and performed approximately 500 laparoscopic colorectal surgeries before the start of this series, and was trained for a certificate in robotic surgery.

The objectives of this program include pre-operative oral fluids, no routine bowel preparation, and no nasogastric tube. All patients had a urinary catheter placed before induction of general anesthesia, which was not routinely left in place for more than 24 h. All patients had an epidural catheter placed for post-operative analgesia. Ceftazidime and metronidazole have been chosen as our prophylactic agents for short-term infections. In addition, early post-operative movement out of bed is what we recommend (10).

### Surgical Procedures

After the pneumoperitoneum was successfully introduced, the curved incision was inserted into the 10 mm port for the laparoscope. At the beginning of the operation, a laparoscope was used to examine the abdomen and locate the tumor. It was also used to introduce the remaining ports. An 8 mm trocar (arm 1) was placed in the left upper abdomen, an 8 mm trocar (arm 2) was established in the lower abdomen, and a 10mm Trocar (auxiliary port) was placed in the anterior axilla line of the left middle abdomen. In terms of surgical techniques, we adopted the complete mesoscopic exception and D3 lymph node dissection (11).

The CUSUM method is a time-weighted control chart method that is increasingly used in studies related to LCs (12). This method calculates the degree of deviation of each sample observation from the target value, and by summing the values, the CUSUM is calculated. Since the CUSUM control chart is cumulative, even minor fluctuations in the process mean can lead to a steady increase (or decrease) in the cumulative deviation value. The formula is as follows:

$$\mathrm{CUSUM}=\overset{n}{\underset{i=1}{\sum }}\left(x_{i}-m\right)$$

CUSUM is proposed to improve the sensitivity of the control chart method. In the CUSUM chart, each value summarizes what is happening, and all the previous points are on the curve, which can add up the small offsets in the process to achieve a magnifying effect (13). Therefore, one of its advantages is that it can detect abnormal fluctuations of data sensitively. This makes CUSUM charts particularly suitable for detecting very small changes (14). Despite its limitations, CUSUM is considered to be one of the best methods in the field of medical research.

*x_i_* represents the actual operative time for each patient, and *m* represents the average operative time for the same group of patients. In this study, the difference (degree of deviation) between the operation time of each patient in the chronological order (sample observation) and the average operation time of the whole group (target value) is summed cumulatively to obtain the LC value.

SPSS 23. 0 was used for the statistical analysis of the data. *T*-test was used to compare measurement data, and a chi-square test was used to compare count data. *P* < 0.05 indicated that the differences were statistically significant.

## Results

We analyzed the primary data of 76 consecutive patients, of whom the mean age of the patients was 61.5 years, 47 were males, and 29 were females. The distribution according to the ASA score was as follows: ASA I 5% (4/76), ASA II 77% (59/76), and ASA III 17% (13/76). Pre-operative tumor staging was, according to the American joint committee on Cancer (AJCC) 8th edition pathological staging Stage I 13% (10/76), Stage II 48% (34/76), and Stage III 42% (32/76) (Table 1).

**Table 1:** Basic patient characteristics.

Patient characteristics	Patient
Age (year)	
Mean ± SD	61.51 ± 12.1
Gender	
Male	61.8% (47/76)
Female	38.2% (29/76)
Body mass index (BMI)	
Mean ± SD	24.01 ± 3.68
ASA score	
I	5.3% (4/76)
II	77.6% (59/76)
III	17.1% (13/76)
Clinical stage	
Stage I	13.2% (10/76)
Stage II	44.7% (34/76)
Stage III	42.1% (32/76)

**Figure 1:**
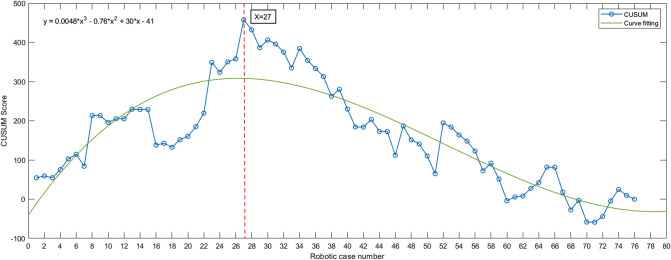
Da Vinci robot-assisted right hemicolectomy—cumulative sum (CUSUM) diagram. The curve slope was steadily negative after surgery in 27 cases, suggesting that 27 cases crossed the learning curve after surgery. Fitted curve formula: *y* = 0.0048*x^3^ – 0.76*x^2 ^+ 30*x – 41.

The CUSUM method curve reaches a maximum of 27 cases and gradually decreases. The curve can be divided into two phases according to the slope of the curve: phase I being the initial learning phase (first 27 cases) and phase II being the proficiency phase (last 49 cases) (Figure 1).

There was no statistical difference between the two stages in terms of general information, including gender, age, BMI, and CCI (Table 2).

**Table 2:** Pre-operative patient characteristics.

Patient characteristics	Phase I	Phase II	*P*-value
	*N* = 27 (patients 1–27)	*N* = 49 (patients 28–76)	
Age (year)			0.686
Mean ± SD	60.81 ± 9.8	61.89 ± 13.2	
Gender			0.402
Male	55.6% (15/27)	65.3% (32/49)	
Female	44.4% (12/27)	34.7% (17/49)	
Body mass index			0.587
Mean ± SD	24.33 ± 3.56	23.85 ± 3.76	
ASA score			0.135
I	11.1% (3/27)	2% (1/49)	
II	66.7% (18/27)	83.7% (41/49)	
II	22.2% (6/27)	14.3 (7/49)	
T-stage			0.301
T1	11.1% (3/27)	4.1% (2/49)	
T2	18.5 (5/27)	8.2% (4/49)	
T3	63 (17/27)	75.5% (37/49)	
T4	7.4% (2/27)	12.2% (6/49)	
N-stage			0.873
N0	55.6% (15/27)	61.2% (30/49)	
N1	28.6% (8/27)	24.5% (12/49)	
N2	14.8% (4/27)	14.3% (7/49)	
Degree of tumor differentiation			0.19
Low	3.7% (1/27)	18.4% (9/49)	
Middle	92.6% (25/27)	77.6% (38/49)	
High	3.7% (1/27)	4% (2/49)	
Charlson Comorbidity Index			0.99
Mean ± SD	3.14 ± 1.65	3.14 ± 1.173	

The mean operative time for all patients was 170.4 min, with negative post-operative margins and no intraoperative conversion to open abdomen cases. We observed that after passing through the initial phase of the LC, the total operative time decreased significantly during the procedure. The operative time and intraoperative bleeding were better for patients in the proficiency phase than in the initial phase in statistically significant.

Gastrointestinal recovery time was measured using the time to first fluid diet, with the meantime to first fluid diet being 5.37 days in the initial phase and 5.55 days in the proficiency phase. The mean length of hospital stay was 10.67 days for the initial group and 8.8 days for the proficiency phase. The number of post-operative lymph nodes detected was 18.4 and 21 in the first and second stages, respectively, and the post-operative tumor diameter was 4.43 and 5.39 inside. There was no statistical difference between the pathological data of the two stages, including tumor diameter, degree of tumor differentiation, T-stage, and N-stage, and the complication of surgery in the initial group was one case of surgical bleeding (conservative treatment). The perioperative complication in the skilled group was adhesive bowel obstruction (re-operation) (Table 3).

**Table 3:** Comprehensive post-operative evaluation indicators.

Outcomes	Phase I	Phase II	*P*-value
	*N* = 27 (patients 1–27)	*N* = 49 (patients 28–76)	
Operative time (min)			0.009
Mean	187.4 ± 45.6	161.1 ± 37.7	
Intraoperative blood loss (ml)			0.031
Mean	170.4 ± 217.2	95.7 ± 72.8	
Post-operative hospital stay (days)			0.415
Mean	10.67 ± 13.11	8.8 ± 5.67	
Lymph node harvested			0.1
Mean	18.4 ± 5	21 ± 7.1	
Days to oral feeding			0.659
	5.37 ± 2.0	5.55 ± 1.51	
Post-operative tumor diameter			0.08
	4.43 ± 1.7	5.39 ± 2.55	
Conversion to laparotomy	0	0	N/A
Post-operative complications	1	1	0.665
Positive resection margins	0	0	N/A
Re-operation within 30 days	0	1	N/A

## Discussion

RARH is an emerging and developing technology that is significantly different from conventional surgery and laparoscopic techniques. The research on the LC of the da Vinci robot-assisted right hemicolectomy will not only guide future procedures but will also be more conducive to the promotion of this technique. The CUSUM method is particularly suitable for observing the slow LC process from quantitative to a qualitative change in skills and requires a small sample size and no grouping, which makes it more practical and accurate (12, 15).

There are no absolute contraindications to robotic surgery. Still, relative contraindications are similar to most laparoscopic procedures, including cardiopulmonary insufficiency, coagulation disorders, severe abdominal adhesions, extensive tumor metastases, and combined pregnancy. When surgeons are in the early stages of the LC, they should selectively choose suitable patients. Ideal candidates for robotic surgery include those with few medical comorbidities, an appropriate BMI, a small primary tumor, and no previous adjuvant chemotherapy (16).

In the retrospective study of Amilcare Parisi, after analyzing the data of 108 cases, they divided the LC into three stages, and 44 cases were needed to pass the first stage of the LC (17). In addition, 23 consecutive cases were included in the Paolo Raimondi study. According to the CUSUM method, they divided the LC into two stages, and 13 cases were needed to cross the first stage (18). According to our experience, the number of cases that have crossed the LC is 27. Through the collection of the above data, we can find that the data of different researchers are very different, which may be related to the number of studies included and the laparoscopic surgery of the surgeons themselves. But what is common is that after passing the surgical curve, the time of operation and the amount of blood loss are relatively reduced. Our analysis of this may be due to the operator's more extensive laparoscopic experience and our use of a more mature external anastomosis, which in combination may have shortened the LC somewhat, which facilitates the spread of the robotic technique.

It has been shown that robotic assistance in radical surgery for right hemicolectomy significantly reduces intraoperative bleeding compared with laparoscopic surgery, decreases the rate of intermediate openings, accelerates post-operative recovery of gastrointestinal function, shortens hospital stay length, and has potential advantages in reducing post-operative complications (19). Our study observed that the number of harvested lymph nodes was similar across the different stages of research and the cut margins were all negative, consistent with the pathology required for radical tumor management. And with the accumulation of surgical experience, we observed less intraoperative bleeding than before, which may be a better suggestive result. In terms of tumor prognosis, relevant small sample studies have shown similar long-term disease-free survival and overall survival rates for robotic vs. laparoscopic surgery (20).

In our study, it took an average of about 5 days to restore diet after the operation, which is longer than that reported in most studies (21). In our post-operative management, patients usually start drinking water on the day of exhaust. But in this study, we counted the time when patients switched to a semi-fluid diet. In fact, people who start drinking water and eating whole-liquid food start earlier than this.

Although the da Vinci robot has many advantages, there are still many difficulties in the early stages of learning. First of all, the pre-operative loading time is extended. Since the Angle and position of the robot arm are relatively fixed, it cannot move freely during the operation. Secondly, the lack of force feedback from the robotic arm prevents the operator from feeling the force feedback from the arm and can only judge the effect of pulling and cutting by sight (22).

This retrospective observational study has some limitations: the number of cases was not significant, only 76; it involved only one surgeon who already had extensive experience with laparoscopic hands; and only one method was used to assess the surgical curve, CUSUM, without taking more than one approach. In the future, we hope to conduct multicenter clinical trials with multiple evaluation criteria involving several surgeons with different experiences to assess the surgical curve of RARH.

## Conclusion

In our experience, the number of cases required for a physician to cross the initial stage to reach proficiency in the robot-assisted hemicolectomy for colon cancer is 27. In addition, the proficient stage takes less time to operate than the initial stage and is associated with relatively less intraoperative bleeding.

## Data Availability

The raw data supporting the conclusions of this article will be made available by the authors, without undue reservation.
